# Pharmacogenomic scores in psychiatry: systematic review of current evidence

**DOI:** 10.1038/s41398-024-02998-6

**Published:** 2024-08-06

**Authors:** Nigussie T. Sharew, Scott R. Clark, K. Oliver Schubert, Azmeraw T. Amare

**Affiliations:** 1 Discipline of Psychiatry, Adelaide Medical School, The University of Adelaide, Adelaide, SA Australia; 2 Asrat Woldeyes Health Science Campus, Debre Berhan University, Debre Berhan, Ethiopia; 3 Division of Mental Health, Northern Adelaide Local Health Network, SA Health, Adelaide, Australia; 4 Headspace Adelaide Early Psychosis - Sonder, Adelaide, SA Australia

**Keywords:** Predictive markers, Schizophrenia

## Abstract

In the past two decades, significant progress has been made in the development of polygenic scores (PGSs). One specific application of PGSs is the development and potential use of pharmacogenomic- scores (PGx-scores) to identify patients who can benefit from a specific medication or are likely to experience side effects. This systematic review comprehensively evaluates published PGx-score studies in psychiatry and provides insights into their potential clinical use and avenues for future development. A systematic literature search was conducted across PubMed, EMBASE, and Web of Science databases until 22 August 2023. This review included fifty-three primary studies, of which the majority (69.8%) were conducted using samples of European ancestry. We found that over 90% of PGx-scores in psychiatry have been developed based on psychiatric and medical diagnoses or trait variants, rather than pharmacogenomic variants. Among these PGx-scores, the polygenic score for schizophrenia (PGS_SCZ_) has been most extensively studied in relation to its impact on treatment outcomes (32 publications). Twenty (62.5%) of these studies suggest that individuals with higher PGS_SCZ_ have negative outcomes from psychotropic treatment — poorer treatment response, higher rates of treatment resistance, more antipsychotic-induced side effects, or more psychiatric hospitalizations, while the remaining studies did not find significant associations. Although PGx-scores alone accounted for at best 5.6% of the variance in treatment outcomes (in schizophrenia treatment resistance), together with clinical variables they explained up to 13.7% (in bipolar lithium response), suggesting that clinical translation might be achieved by including PGx-scores in multivariable models. In conclusion, our literature review found that there are still very few studies developing PGx-scores using pharmacogenomic variants. Research with larger and diverse populations is required to develop clinically relevant PGx-scores, using biology-informed and multi-phenotypic polygenic scoring approaches, as well as by integrating clinical variables with these scores to facilitate their translation to psychiatric practice.

## Introduction

Psychiatric disorders are significant contributors to the global disease burden and represent a major public health concern [1], highlighting the urgent need for effective prevention and treatment strategies [2]. The 2022 World Health Organization (WHO) report estimates that nearly a billion people suffer from psychiatric disorders, with an associated economic loss of $2 trillion per year and this figure is expected to rise to $6 trillion by 2030 [3–6].

Pharmacological treatments including antidepressants, antipsychotics, mood stabilizers, and anxiolytics are commonly prescribed for people suffering from psychiatric disorders [7]. However, the effectiveness of these medications varies between individuals, with some responding well while others do not show notable improvement or experience adverse effects [7]. For example, among patients with major depressive disorder (MDD), 30–40% fail to respond to the first-line pharmacological treatment options of selective serotonin reuptake inhibitors (SSRIs), and 10-–45% exhibit moderate to severe treatment-related side effects [8, 9]. Similarly, only 30% of patients with bipolar disorder (BD) show a full clinical response to first-line lithium monotherapy [10], and up to 25% of patients with first-episode schizophrenia (SCZ) are treatment-resistant to first-line antipsychotics [11]. This variability in pharmacological treatment outcomes can be attributed to the complex interplay of genetic and environmental factors, including patients’ clinical characteristics (e.g., severity, number, and duration of illness episodes), as well as sociodemographic variables [12]. For example, in individuals with MDD, genetic factors account for 42–52% of the observed differences in antidepressant treatment response, while environmental factors contribute to the remainder [13, 14].

To date, studies employing both candidate gene investigations (pharmaco*genetics*) and genome-wide (pharmaco*genomic*) approaches, have successfully pinpointed genetic variations associated with treatment outcomes in psychiatry, including response [15], remission [16], resistance [17] and adverse drug reactions [18]. For instance, the pharmaco*genetic* approach has uncovered genetic polymorphisms within genes encoding drug-metabolizing enzymes including those involved in the metabolism of various psychotropic drugs (e.g., *CYP2D6* and *CYP2C19*) [19] as well as drug transporters (e.g., *5-HTTLPR)*, establishing their association with patients’ responses to medications [20]. This evidence is now incorporated into commercially available pharmacogenetic testing panels, aiding drug selection and dose adjustments and ultimately aiming at improving medication efficacy and tolerability [21, 22]. Similarly, the pharmaco*genomics* approach has revealed a number of genetic polymorphisms located within or near pharmacologically relevant candidate genes that influence individuals’ reaction to psychiatric medications [10]. For instance, Hou et al. identified four linked genetic variants on chromosome 21 associated with lithium response in a Genome-wide Association Study (GWAS) [10]. It has been challenging, however, to translate these pharmaco*genomic* findings into clinical practice, mainly due to the small effect size of individual genetic variants on treatment outcomes, along with a limited understanding of gene function [23].

In an effort to improve effect estimates and make pharmaco*genomic* findings more clinically relevant, researchers have recently adopted polygenic score methods that combine the effect of multiple genetic variants across the genome and have developed pharmacogenomic scores (PGx-scores) [24, 25]. In this systematic review, we provide a detailed account of the research undertaken to date, and of the performance, shortfalls, and future recommendations for the development of PGx-scores for the personalisation of psychiatric care.

## Methods

This systematic review adhered to the PRISMA updated guidelines 2020 [26] and was registered with the International Prospective Register of Systematic Reviews (PROSPERO) on February 9, 2023 (ID = CRD42023395404). The review protocol was prepared before commencement to ensure a transparent and standardized methodology.

## Search strategy, inclusion, and exclusion criteria

The literature search was performed across three databases including PubMed, EMBASE, and Web of Science databases from January 1^st^, 2005 to 22^nd^ August 2023, by using search string: ((“Polygenic score*“ OR “Polygenic risk score*“ OR “Risk profile score*“ OR “Genetic risk score*“ OR “Gene score*“ OR “Genetic score*“ OR polygenic* OR “Pharmacogenomic variants” OR “Pharmacogenomic testing” OR Pharmaco-omic* OR pharmacogeno* OR “Pharmacogenetics”) AND (“Antipsychotic agents” OR antipsycho* OR “Antidepressive agents” Antidepress* OR “Anti-anxiety agents” OR Anti-anxiet* OR Valproic acid OR Valproate OR Divalproate OR Divalproex OR Carbamazepine OR Oxcarbazepine OR Risperidone OR Gabapentin OR Lamotrigine OR Licarbazepine OR Pregabalin OR Tiagabine OR Zonisamide OR Lithium)).

Our search strategy included all original studies that developed PGx-score for drug-related phenotypes such as, drug dosage, therapeutic drug response, resistance, drug-induced side-effects, relapse or hospitalisation in psychiatry. We included studies that reported weighted PGx-score for the drug-related phenotypes mentioned above while excluding publications in languages other than English, conference abstracts, case reports, editorials, notes, and systematic reviews. NTS screened the studies for inclusion under the supervision of ATA. In the final step, all studies were imported into Endnote version 20, a reference manager software. Duplicate entries were removed, and the selection of studies was carried out based on the predetermined inclusion and exclusion criteria. Supplementary Table 1 provides details of the systematic search strategies and results in each database.

## Data extraction and synthesis

NTS extracted data using a customised data extraction excel sheet format, under the supervision of ATA. This excel sheet included information on the authors’ characteristics, details of the drug outcomes, characteristics of the study cohort (such as base, target, and validation cohorts), number of variants included in the polygenic score (PGS), polygenic scoring methods, and association effect estimates. We summarized the extracted data in the supplementary table 2.

The “target cohorts” describe the cohorts where the PGS was developed and tested, while “discovery cohorts” refer to the cohorts utilized to create GWAS summary statistics. “Validation cohorts” are independent cohorts where the PGSs were validated. “Variance explained” measures the proportion of phenotype variance in which the PGS can account for in a predictive model assuming linear effects. Coefficient of effect estimates, standard error, and sample size were used to calculate odd ratios if not reported in the studies.

The results were organized thematically based on the psychiatric disorders that were studied, as well as the specific phenotypes investigated, including treatment response, treatment resistance, and drug-induced side effects. Supplementary Table 3 provides the definitions and detailed description of each study’s treatment outcome.

## Quality assessment

The quality of included studies was assessed using a quality assessment form adapted from previously validated and published sources [27, 28]. The assessment criteria covered various aspects of the study design, such as the rationale and methods of PGS, power calculation, inclusion and exclusion criteria, basic characteristics of the study population, availability of validation cohort, type of analysis, correction for multiple testing, and consideration of confounders in the analysis. The quality assessment was conducted by NTS under the supervision of ATA.

## Results

**Fig. 1:**
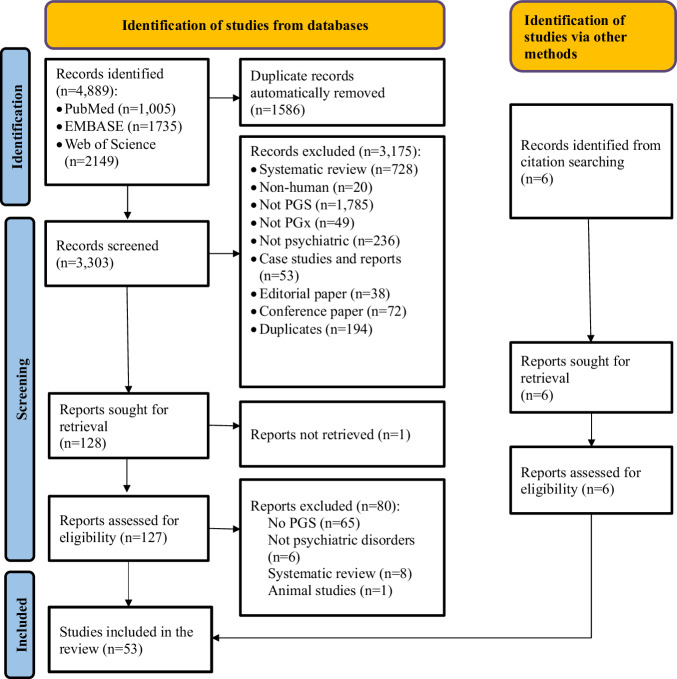
PRISMA flow diagram showing the steps of screening studies included in this systematic review. PGS Polygenic score, PGx Pharmacogenomics.

Our initial search identified a total of 4889 studies that were potentially relevant to the research topic. After removing 1586 duplicated publications, 3,303 articles remained for the title and abstract screening. Subsequently, 3175 studies were excluded during the initial title and abstract screening phase, leaving 127 articles for full-text review. Finally, 53 studies met the predetermined inclusion criteria and were included in the final synthesis. Figure 1 presents the flowchart of the step-by-step process of study selection with reasons for exclusion.

## Quality assessment

Nearly three-quarters (39/53) of studies described the rationale for the selected polygenic scoring methods, and about 20% (10/53) of studies performed a power calculation. All studies reported the inclusion and exclusion criteria for participants’ selection. Only fourteen studies used external cohorts to validate their findings. Correction for multiple testing was performed in 83.2% (44/53) of studies. Detailed results of the quality assessment are provided in the supplementary table 4.

**Fig. 2:**
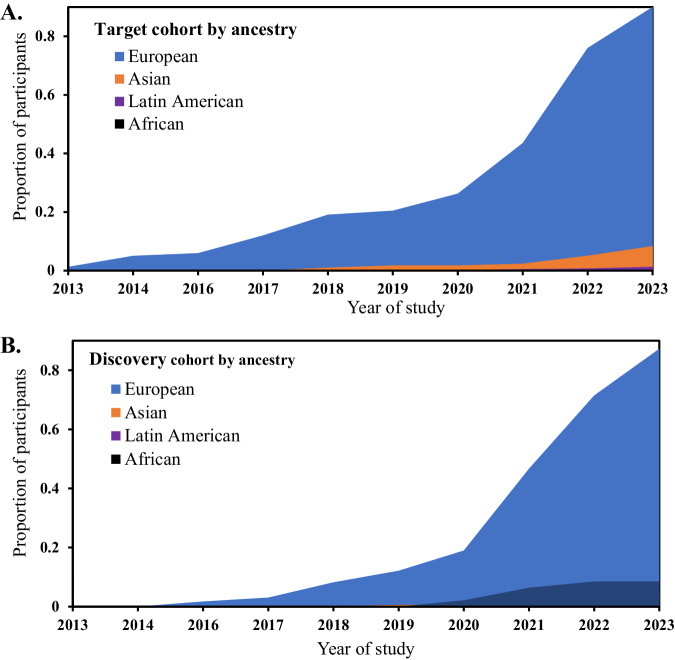
Ancestry characteristics of study participants in the reviewed articles from 2013 to 2023. **A** Target cohort; **B** Discovery cohort.

Most studies 37 (69.8%) were conducted on samples comprising individuals of European ancestry. Eleven studies (20.8%) included participants from other ancestries, such as African, African American and/or East Asian. Three studies targeted only Latin American participants and another two studies were conducted specifically on samples of East Asian ancestry. However, there was no study solely centred on samples of African ancestry. A combined analysis of both the target and discovery samples showed that 14,893,321 (90%) of participants had European descent, with an increased trend over the years 2013–2023, both in the target (Fig. 2A) and discovery cohorts (Fig. 2B).

The sample sizes across the studies varied widely, ranging from 44 participants [29] to 12,863 participants [30] with a median sample size of 863 in the target cohorts. Three major psychiatric conditions, namely SCZ, MDD, and BD, were the focus of the included studies. In the case of SCZ, nearly 80% (21/27) of studies investigated the association between PGS and response to second-generation antipsychotics (clozapine, risperidone, lurasidone, olanzapine, aripiprazole, quetiapine, ziprasidone, and perphenazine). About half of SCZ studies (13/27) exclusively analysed clozapine treatment outcome. Nearly three-quarters of the included studies involving patients with MDD (14/19) considered the relationship between PGS and SSRIs such as citalopram or escitalopram. Six out of seven included studies of patients with BD developed PGx-scores and examined their associations with lithium treatment response.

The characteristics of the included studies and summary of the findings are presented in Tables 1–3 and described in the following sections.

**Table 1:** Summary of findings on the association between PGx-score and antipsychotic treatment outcomes in patients with schizophrenia.

Author	Medication studied	PGSs for	Target cohort and treatment outcome			Main association findings	
			*Cohort*	*N*	*Treatment outcome*	*Effect estimates [OR/RR (95% CI)]*	*R*^2^ *(%)*
Guo et al. [35]	SGAs	SCZ	CAPOC and CAPEC	3686	Response	Correlation between PGS_SCZ_ and treatment response = -0.05[-0.09, -0.01]	0.51
Okhuijsen-Pfeifer et al. [38]	Clozapine	SCZ	CLOZIN	684	Response	Highest PGS_SCZ_ tertile vs lowest tertile: 1.94 [1.33–2.81]	1.85
Santoro et al. [44]	Risperidone	SCZ	CAISM	60	Response	Higher PGS_SCZ_ associated with reduced depressive symptoms.	0.19
Zhang et al. [42]	Risperidone or olanzapine	SCZ	ZHH-FE	77	Response	PGS_SCZ_ associated with having worse 12 week symptom scores, OR = 1.43	3.24
Li et al. [45]	Lurasidone	SCZ	Registered patients	302	Response	PGS_SCZ_ was associated with the exacerbation of positive symptoms of SCZ.	ROC = 79.20%
Hettige et al. [48]	SGAs	SCZ	CAMH	83	Response	NSA	NA
Kappel et al. [34]	Clozapine	SCZ	CLOZUK	3133	Clozapine dosage	PGS_SCZ_ positively correlated with increased clozapine dose; 12.21[4.81,19.62]	0.32
Lin et al. [33]	Clozapine	SCZ	GROUP and CLOZIN	2505 and 687	Likelihood of clozapine prescription	Higher PGS_SCZ_ with lower group of individuals taking clozapine: RR, 3.24[2.76,3.81]	2.59
O’Connell et al. [54]	Clozapine	SCZ, TRS and BMI	TDM	1733	TRS	NR	5.62 by the shared variants of TRS and BMI
Talarico et al. [36]	SGAs	SCZ	PROESQ	174	TRS	Top three PGS_SCZ_ deciles versus bottom three PGS_SCZ_ deciles: 2.42 [1.35, 3.49]	~2.00
Gasse et al. [43]	Clozapine	SCZ	DCRS	593	TRS	TRS associated with one SD increase in PGS_SCZ_, HR = 1.11 [95% CI; 1.00–1.24].	NR
Werner et al. [40]	FGAs and SGAs	SCZ	TOP	321	TRS	Higher PGS_SCZ_ vs lower: 1.5 [1.13–1.96]	1.70
Pardinas et al. [37]	Clozapine	SCZ	Cardiff COGS and STRATA-G	817	TRS	Higher PGS_SCZ_ vs lower PGS_SCZ_: 1.22 [1.05–1.41]	2.03
Wimberley et al. [49]	Clozapine	SCZ	DPR	862	TRS	NSA	NA
Martin & Mowry [50]	Clozapine	SCZ	MGS	612	TRS	NSA	NA
Kowalec et al. [51]	Clozapine	SCZ, MDD and BD	SNPD	4936	TRS	NSA	NA
Facal et al. [39]	Clozapine	SCZ	GEHRS	1241	Hospitalisation	Higher PGS_SCZ_ with lower PGS_SCZ_: 1.48, 95% CI [1.10-1.97]	2.70
Mayen-Lobo et al. [29]	Clozapine	SCZ, MDD and BD	NINN	44	Clozapine metabolic ratio	PGS_BD_ was associated with the Clozapine metabolic ratio and no significant association with SCZ and MDD	0.21
Blackman et al. [53]	FGAs	Cognitive ability	Healthy volunteers	71	Cognitive symptom	Medication-related performance changes in category fluency positively associated with PGScog.	0.27
Yoshida et al. [31]	SGAs	SCZ and T1D	CATIE and CAMH	151 and 138	AIWG	Higher PGS for T1D compared with lower: 4.67[1.66,13.12]; Higher PGS_SCZ_ compared with lower: 2.04[1.14,3.66]	~0.06
Muntane et al. [32]	FGAs and SGAs	SCZ and BMI	PAFIP	381	AIWG	Higher PGS for BMI and SCZ vs lower: OR (1.33)	0.02
Morgenroth et al. [52]	Clozapine	SCZ and OCD	CLOZIN	102	Clozapine-induced OCD	NSA	NA
Segura et al. [55]	FGAs and SGAs	SCZ, BD, depression and T2D	CIBERSAM	231	Antipsychotic-induced metabolic dysregulation	PGS for TC, TG and LDL were significantly associated with corresponding metabolic parameters.	~1.20–4.30
Hommers et al. [47]	FGAs and SGAs	QT intervals	RPUHW	804	QT prolongation	Higher PGS for QT interval vs lower: 1.19 [1.02, 1.44]	NR
Lu et al. [46]	SGAs	Myocardial infarction	CAPOC	2040	QTc prolongation	Higher PGS for myocardial infarction was associated with increased antipsychotic induced QTc interval prolongation	~0.01
Maciukiewicz et al. [127]	FGAs and SGAs	BMI and obesity	CAMH	201	AIWG	NSA	NA
Lacaze et al. [41]	Clozapine	Myocarditis	Registered patients	109	Clozapine induced myocarditis	NR	ROC = 66%

*PGx-score* Pharmacogenomic polygenic score, *PGS* Polygenic score, *FGAs* first-generation antipsychotics, *SGAs* second-generation antipsychotics, *SCZ* Schizophrenia, *MDD* Major Depressive Disorders, *MDE* Major Depressive Episode, *BD* Bipolar Disorders, *ADHD* Attention Deficit Hyperactivity Disorders, *OCD* Obsessive-Compulsive Disorders, *PGS*_*SCZ*_ PGS for SCZ, *PGS*_*MDD*_ PGS for MDD, *PGS*_*BD*_ PGS for BD, *CAPOC* Chinese Antipsychotics Pharmacogenomics Consortium, *CAPEC* Chinese Antipsychotics Pharmacogenetics Consortium, *CLOZUK* Genome-wide genotype information for SCZ cases from the UK, *CAISM* Centro de Atenção Integral a Saúde Mental, *PROESQ* Schizophrenia Program at the Universidade Federal de São Paulo, *ZHH-FE* Zucker Hillside Hospital First Episode schizophrenia trial, *DCRS* Danish Civil Registration System, *CLOZIN* Clozapine International, *GROUP* Genetic Risk and Outcome of Psychosis, *TOP* Thematically Organized Psychosis, *Cardiff COGS* Cardiff Cognition in Schizophrenia, *STRATA-G* Genetics Workstream of the Schizophrenia Treatment Resistance and Therapeutic Advances, *CAMH* Centre for Addiction and Mental Health, *PAFIP* Cantabria program for early interventions in psychosis, *CATIE* Clinical Antipsychotic Trails of Interventions Effectiveness, *TDM* Therapeutic drug monitoring, *DPR* Danish population-based registers, *MGS* Molecular Genetics of Schizophrenia, *RPUHW* Registered patients at the University Hospital of Würzburg, *CIBERSAM* Centro de Investigación Biomédica en Red de Salud Mental, *IQ* Intelligent Quotient, *proxyDNAm* Proxy DNA methylation, *PGS*_*cog*_ Polygenic score for cognitive ability, *TRS* Treatment resistant Schizophrenia, *SNPD* Swedish National Prescribed Drug Register, *BMI* Body Mass Index, *HDL* High-density lipoprotein, *LDL* Low-density lipoprotein, *TG* Triglyceride, *TC* Total cholesterol, *T1D* Type 1 Diabetes, *T2D* Type 2 Diabetes, *ADRs* Adverse Drug Reactions, *HR* Hazard Ratio, *ROC* Receiver Operating Characteristics curve, *SD* Standard Deviation, *NSA* No significant association, *NR* Not reported, *NA* Not applicable, *CI* Confidence Interval.

## The association of pharmacogenomics scores with antipsychotics treatment outcomes

**Fig. 3:**
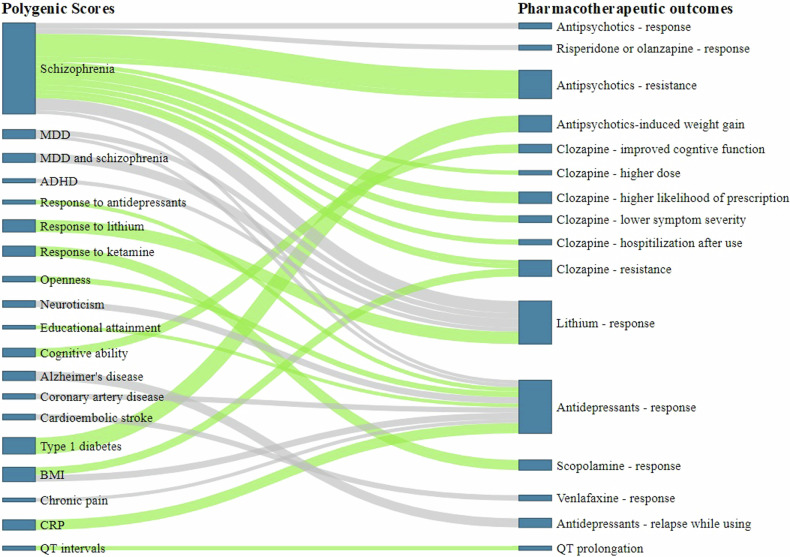
Figure showing the relationship between different pharmacogenomic scores for different traits and pharmacotherapeutic outcomes in psychiatry. Green line represents the positive associations of pharmacogenomic scores with treatment outcomes; Gray line indicates negative associations between PGx-scores and treatment outcomes. A wider (thick) line represents a stronger association. MDD Major depressive disorders, ADHD Attention Deficit Hyperactivity Disorders, BMI Body mass index, CRP C-reactive Protein.

Of all PGx-scores reviewed, the polygenic loading for schizophrenia (PGS_SCZ_) has been extensively studied (22 publications) in relation to its influence on antipsychotics treatment outcomes (see Table 1 and Fig. 3). Among these, 17 studies revealed that individuals with a higher genetic load for SCZ had a poorer treatment outcome to antipsychotics [31–47] while the remaining studies did not find significant associations [48–52]. For example, in patients with SCZ, a negative correlation (*r* = -0.05 [95%CI: -0.09– -0.01]) was found between PGS_SCZ_ and response to second-generation antipsychotics (olanzapine, aripiprazole, risperidone, quetiapine, haloperidol, ziprasidone, perphenazine) following 6 weeks treatment [35]. Kappel et al. [34] observed a positive correlation (*β* = 12.21; 95%CI: 4.81–19.62) between PGS_SCZ_ and high clozapine dosing (>600 mg/day), suggesting that individuals with a higher PGS_SCZ_ may require increased doses of clozapine to achieve effective treatment response [34]. The patients with a higher PGS_SCZ_ were observed to have a 3.24 times higher likelihood of having a clozapine prescription compared with healthy control (RR = 3.24 [95%CI:2.76–3.81]), indicating the clinical relevance of using PGS_SCZ_ to personalize psychotic treatment [33]. In patients treated with risperidone, those who had a higher PGS_SCZ_ reported more depressive symptoms [44], and worsened positive and negative psychotic symptoms [45]. A higher PGS_SCZ_ was associated with a poor response to olanzapine or risperidone OR = 1.43 [95%CI:1.19–1.67] [42] and an increase of one standard deviation in PGS_SCZ_ was associated with an ~11% increase in the risk of developing treatment-resistant schizophrenia (TRS) (OR = 1.11 [95%CI: 1.00–1.24]) [43].

Moreover, a PGS_SCZ_ was associated with antipsychotic treatment resistance and side-effects in patients with SCZ. Specifically, patients with a higher polygenic load for SCZ were 1.22 times [95%CI: 1.05–1.41; *R*^*2*^ = 2.03%] more likely to be resistant to clozapine [37], had 1.50 times [95%CI: 1.13–1.96; *R*^*2*^ = 1.70%] higher odds to experience resistance to other antipsychotics [40] and were more likely to develop antipsychotic-induced weight gain (AIWG) [31, 32].

In decile-based comparisons, patients in the top three PGS_SCZ_ deciles had a 2.42 [95%CI: 1.35–3.49; *R*^*2*^ ~ 2.00%] times higher odds of poor response to various antipsychotic medications (olanzapine, risperidone, quetiapine, and clozapine) [36] and the odds of treatment resistance for those in the 8th PGS_SCZ_ decile was 6.50 times [95%CI: 1.47–28.80] higher than for patients in the 1st decile [36]. Patients with a higher PGS_SCZ_ had 1.48 times [95%CI: 1.10–1.97; *R*^*2*^ = 2.70%] higher odds of psychiatric hospitalizations and were hospitalized longer [39]. Interestingly, in a study by Okhuijsen-Pfeifer et al. [38], patients treated with clozapine who were in the highest PGS_SCZ_ tertile group were 1.94-fold more likely to experience low (i.e., more favourable) symptom severity [95%CI: 1.33–2.81; *R*^*2*^ = 1.85%], compared to those in the lowest PGS_SCZ_ tertile group [38].

Additionally, the polygenic scores for body mass index (BMI), cognitive function, BD, and cardiometabolic traits have been assessed for their potential to predict antipsychotic treatment outcomes. These studies showed statistically significant associations. For example, a higher genetic loading for general cognitive ability was associated with better cognitive function following antipsychotic treatment [53]. Patients with SCZ carrying a greater genetic load for higher BMI were at a higher risk of being resistant to clozapine treatment [54], while those with a higher genetic load for myocarditis were more likely to develop clozapine-induced myocarditis [41] and a higher genetic loading for myocardial infarction was associated with increased antipsychotic-induced QTc interval prolongation [46]. The higher genetic loading for QT interval was also significantly associated with QT interval prolongation in schizophrenic patients taking antipsychotics [47]. The polygenic loading for BD (PGS_BD_) was also found to be significantly associated with clozapine metabolic ratio [29]: a measure of how clozapine is metabolized within the body, which may impact treatment response or adverse effects. In patients with first-episode psychosis, higher genetic loadings for HDL, LDL, and total cholesterol predicted antipsychotic-induced metabolic disturbance [55].

It is important to note that in the majority of studies, polygenic scores were developed using disease-specific genetic variants, and pharmacogenomic variants were considered in only a few studies. Using pharmacogenomic variants, O’Connell and colleagues developed a PGx-score for *clozapine resistance* which was significantly associated with TRS, accounting for ~5.0% of the variance [54]. Table 1 provides a summary of the association findings between PGx-score and antipsychotic treatment outcomes in patients with schizophrenia.

## The association of pharmacogenomic scores with antidepressants treatment outcomes

In patients with MDD, studies have revealed the association of polygenic scores for psychiatric disorders, personality traits, and physical illnesses with antidepressant treatment outcomes (see Table 2 and Fig. 3). For instance, a study by Pain et al. identified that a higher PGS_SCZ_ has been associated with poorer response to antidepressants (OR = 0.97 [95%CI: 0.96–0.98; *R*^*2*^ ~ 0.01%]) [56]. In a study by the Genome-Based Therapeutic Drugs for Depression (GENDEP) investigators, the polygenic loading for MDD (PGS_MDD_) was significantly associated with response and remission to SSRIs and tricyclic antidepressants (TCAs) treatment, although the direction of association was not reported [57]. A study that assessed the relationship between PGS for various personality traits and response to SSRIs (citalopram, escitalopram, fluvoxamine) [58] found that a higher genetic loading for openness personality trait was associated with a better SSRIs treatment response after 8 weeks of treatment OR = 1.58 [95%CI, 1.10–2.90] while the PGS for neuroticism was negatively associated with SSRIs treatment response [58]. The negative association between PGS for neuroticism and antidepressant treatment outcome was also reported in Ward et al's study [59]. Genetic loading for cardiometabolic diseases has also shown associations with response to antidepressant treatment: Marshe et al. [60] used a PGS for cardioembolic stroke to predict response to venlafaxine, an antidepressant of the serotonin-norepinephrine reuptake inhibitors (SNRI) class, after 12 week treatment. They found that a one standard deviation increase in PGS for cardioembolic stroke was associated with a decreased probability of remission (OR = 0.63 [95%CI:0.48 to 0.83]) or worsened disease symptoms (Montgomery-Asberg Depression Rating Scale (MADRS), β = -5.51 [95%CI: -9.45– -1.57]) [60]. In a different study, individuals with the highest PGSs for coronary artery disease (4^th^ quartile) had 0.53 times ([95%CI, 0.35–0.81]) less likelihood of experiencing favourable response to SSRIs (citalopram, escitalopram, fluvoxamine) compared to those in the 1^st^ quartile [61]. Similarly, those with higher genetic loading for obesity (4th quartile) had a 0.53 times ([95%CI, 0.32–0.88]) lower likelihood of achieving a positive response to SSRIs treatment [61]. In individuals with MDD (*n* = 5218) treated with SSRIs (citalopram, escitalopram) or a TCA (nortriptyline), the PGS for educational attainment was positively associated with SSRI response [56]. In a cohort of patients with psychotic depression treated with sertraline and olanzapine for 36 weeks, those who had a higher polygenic loading for Alzheimer’s disease had a decreased likelihood of relapse (OR = 0.38; [95%CI: 0.18–0.80]) during the study period [62]. Higher PGS for chronic pain was negatively associated with treatment response to SSRIs, TCAs (mirtazapine), and SNRIs (desvenlafaxine) (OR = 0.95 [95%CI: 0.92–0.98]) [30], while a higher PGS for C-reactive protein (CRP) was associated with a better response to escitalopram (OR = 2.92 [95%CI: 1.30–6.49]), but worse response to nortriptyline [63]. Despite the reported significant association between PGx-scores for psychiatric conditions, Nohr et al. [64], Garcia-Gonzalez et al. [65], Li et al. [66], and Tansey et al. [67] did not find any significant association.

**Table 2:** Summary of findings on the association between PGx-score and antidepressant treatment outcomes in patients with major depressive disorders.

Author	Medication studied	PGSs for	Target cohort and treatment outcome			Main association findings	
			*Cohort*	*N*	*Treatment outcome*	*Effect estimates [OR/RR (95% CI)]*	*R2 (%)*
Pain et al. [56]	SSRIs	SCZ and educational attainment	STAR*D, GSRD, GENDEP, DAST, PGRN-AMPS, GENPOD, PFZ, Mayo, GSK, GODS, Miaoli, Taipei, Japan	5117	Response	Higher PGS_SCZ_ with lower: 0.97[0.96,0.98] Higher PGS for educational attainment with lower: 1.02[1.01,1.03]	~0.10
Ward et al. [59]	SSRIs	MDD and neuroticism	PGRN-AMPS and GENDEP	1065	Response	Higher PGS for neuroticism group vs lower group: 0.84, [0.78,0.97] Higher PGS_MDD_ vs lower: 0.98, [0.97,0.99]	NR
Gendep Invesigators et al. [57]	SSRIs	MDD	GENDEP, STAR*D and MARS	2256	Response and remission	NR	0.50-1.20
Nøhr et al. (2022) [64]	Vortioxetine	MDD, BD, SCZ	Seven clinical trials	1364	Response	NSA	NA
Amare et al. [61]	SSRIs	CAD and Obesity	STAR*D and ISPC	865	Response	Fourth PGS for CAD quartile: first in ISPC: 0.71, [0.52-0.96] Fourth PGS for obesity quartile: first in ISPC: 0.53, [0.32–0.88]	CAD = 1.30 Obesity = 0.80
Men et al. [62]	Sertraline and Olanzapine	Antidepressant response and Alzheimer’s disease	STOP-PD II	205	Remission and relapse	PGS for antidepressants symptom improvement with remission status: 1.95[1.20,3.17] PGS for Alzheimer’s disease with relapse: 0.38[0.18,0.80].	NR
Garcia-Gonzalez et al. [65]	SSRIs	SCZ and MDD	GENDEP, STAR*D, GENPOD, GODS, GSK, Pfizer, Muenster	3746	Response	NSA	NA
Li et al. [66]	Esketamine	SCZ, MDD and BD	SUSTAIN-2 and TRANSFORM-3	527	Response	NSA	NA
Tansey et al. [67]	SSRIs	BD	NEWMEDS & STAR*D	2897	Response	NSA	NA
Marshe et al. [60]	Venlafaxine	MDD, Alzheimer’s disease and cardioembolic disease	IRL-GRAY	355	Late-life treatment response	Higher PGS for cardioembolic stroke with non-remission vs lower: 0.63 [0.48, 0.83]	0.46
Fanelli et al. [101]	SSRIs	SCZ, BD, MDD and neuroticism	Brescia, GSRD, Münster, STAR*D, Tartu	3637	Non-response	Higher PGS_MDD_ vs lower: 1.10 [1.02–1.19] (Nominally)	0.24
			Same cohorts	3184	Non-remission	Higher PGS_MDD_ vs lower: 1.14 [1.04–1.24] (Nominally)	0.57
Guo et al. [69]	Ketamine and scopolamine	Scopolamine response	Registered patients with MDD, BP and MDE	127	Response	Higher PGS for scopolamine response vs lower: 2.89 [1.13, 4.32]	6.0%
Meijs et al. [68]	SSRIs	Antidepressants response	ZNA and iSPOT-D	1123	Response	Higher PGS for antidepressants response vs lower: OR (1.18)	2.91
Campos et al. [30]	SSRIs	Chronic pain	AGDS	12,863	Response	Higher PGS_Pain_ group vs lower: 0.95 [0.92, 0.98]	NR
Zwicker et al. [63]	Escitalopram and Nortriptyline	CRP	GENDEP	755	Response	Higher PGS for CRP with lower: 2.91, [1.29, 6.49]	NR
Amare, et al. [58]	SSRIs	Personality traits	PGRN-AMPS and ISPC	1394	Response and symptom remission	Higher PGs for openness vs lower: 1.58, [1.10–2.90]	1.50
Fanelli et al. [103]	SSRIs	SCZ, BD, MDD and neuroticism	GSRD	1148	Treatment non-response	Higher PGS_SCZ_ vs the lower: 2.23: [1.21–4.10] and no significant association with BD, MDD and neurotocism	1.60
Taylor et al. [102]	Mix of antidepressants	MDD	Bethlem Royal Hospital	240	Treatment resistance	NSA	NA
Wigmore et al. [104]	Mixed antidepressants	SCZ, MDD, and BD	GS: SFHS	3452	Treatment resistance	PGS_MDD_ = 1.01; PGS_SCZ_ = 1.01; PGS_BD_ = 1.01 (Nominally)	< 0.01

*PGx-score* Pharmacogenomic polygenic score, *PGS* Polygenic score, *SGAs* Second-generation antipsychotics, *SCZ* Schizophrenia, *MDD* Major Depressive Disorders, *MDE* Major Depressive Episode, *BD* Bipolar Disorders, *ADHD* Attention Deficit Hyperactivity Disorders, *OCD* Obsessive-Compulsive Disorders, *PGS*_*SCZ*_ PGS for SCZ, *PGS*_*MDD*_ PGS for MDD, DCRS Danish Civil Registration System, *PGS*_*Painb*_Polygenic score for pain, PGS_cog_ Polygenic score for cognitive ability, *GSRD* Group for the Study of Resistant Depression, *GENDEP* Genome Based Therapeutic Drugs for Depression, *DAST* Depression and Sequence of Treatment, *PGRN-AMPS* Pharmacogenomics Research Network Antidepressant Medication Pharmacogenomic Study, *GENPOD* Genetics and clinical Predictors of treatment response in depression, *GODS* Geneva Outpatient Depression Study, *PAFIP* Cantabria program for early interventions in psychosis, *STOP-PD II* Study of pharmacotherapy of psychotic depression II, *GEHRS* Galician electronic health records system, *MARS* Munich Antidepressants Response Signature, *CATIE* Clinical Antipsychotic Trails of Interventions Effectiveness, *TDM* Therapeutic drug monitoring, STAR*D Sequenced Treatment Alternatives to Relieve Depression, *AGDS* Australian Genetics of Depression Study, *IRL-GREY* Incomplete Response in Late Life Depression; Getting to Remission, *CRP* C-reactive protein, *CAD* Coronary Artery Diseases, *SSRIs* Selective Serotonin Reuptake Inhibitors, *GSK* Glaxo Smith Kline, *PFZ* Pfizer, *ISPC* International SSRI Pharmacogenomics Consortium, ZNA Ziekenhuis Netwerk Antwerpen, *iSPOT-D* International Study to Predict Optimized Treatment in Depression, *GS-SFHS* Generation Scotland: the Scottish Family Health Study, *SUSTAIN-2* Long-term Safety and Efficacy Study of Intranasal Esketamine in Treatment-resistant Depression, *TRANSFORM* Safety and Tolerability of Intranasal Esketamine Plus Oral Antidepressant in Elderly Participants with Treatment-resistant Depression, *NEWMEDS* New Medications in Depression and Schizophrenia, *SD* Standard Deviation, *NSA* No significant association, *NR* Not reported, *NA* Not applicable, *CI* Confidence Interval.

In contrast to the above studies in which PGx-scores were developed based on diseases or related phenotype-specific variants to predict antidepressants treatment outcomes, a few recent studies used pharmacogenomic variants to calculate PGx-scores, directly indexing treatment outcome phenotypes. In patients with psychotic depression treated with sertraline and olanzapine, those with a higher genetic loading for *antidepressant remission and response* had 1.95 times [95%CI: 1.20–3.17] higher odds of reaching remission after 36 weeks [62]. In a similar context, PGS for *response to SSRIs* (escitalopram, sertraline, venlafaxine) predicted antidepressant treatment response in patients with MDD [68]. A study by Guo et al. [69] utilized variants ranked by their strength of association with ketamine response, a glutamate-modulating antidepressant used in patients with Treatment-Resistant Depression (TRD), to predict scopolamine treatment response in patients with either MDD or BD who had a current major depressive episode [69]. Findings indicated that patients with higher genetic loadings for *ketamine response* had better responses to scopolamine, an emerging antidepressant with effects on acetylcholine (Ach) neurotransmission [69]. Table 2 provides a summary of the pharmacogenomics score with antidepressants treatment outcomes in patients with major depressive disorders.

## The association of pharmacogenomic scores with lithium treatment response

Studies have also found the association of polygenic scores for psychiatric disorders or related phenotypes with lithium treatment response in patients with BD (see Table 3 and Fig. 3*)*. For example, those with a low PGS_MDD_ (first decile) were 1.54 times [95%CI: 1.18–2.01; *R*^*2*^ = 0.91%] more likely to respond favourably to lithium than those who had high MDD genetic loading (10th decile) [70]. A study by Amare et al. showed that a higher PGS_SCZ_ was associated with poorer response to lithium (OR = 0.29 [95%CI: 0.12–0.70; *R*^*2*^ = 0.80]) [71]. Similarly, a higher PGS for ADHD was associated with an unfavourable lithium response (OR = 0.86 [95%CI: 0.77–0.95], *R*^*2*^ = 0.18) [72]. Further studies using the same dataset have shown that a combined analysis of the PGSs of multiple phenotypes and PGS with patients’ clinical data can improve the predictive capacity of polygenic models. For example, a meta-analysis of the association results of the PGS_SCZ_ and PGS_MDD_ provided improved response prediction compared to single disorder PGS [73]. By applying machine learning methods, the PGS_SCZ_ and PGS_MDD_ were combined with clinical data, which resulted in an explained variance of 13.7% in lithium treatment response [74]. In a recent study, lithium clearance, an essential parameter for maintaining therapeutic levels of lithium and adjusting dosage, was positively associated with the PGSs for BMI and estimated glomerular filtration rate (eGFR), while it was negatively associated with the PGSs blood urea nitrogen (BUN) [75]. In addition to the disease-specific polygenic scores mentioned above, a PGx-score was developed for the ConLi^+^Gen sample using pharmacogenomic variants of lithium response (Li^+^R_PGS_), which was then evaluated in both a hold-out subsample and a smaller independent replication cohort. This analysis revealed that individuals in the highest Li^+^R_PGS_ decile were 3.47 times [95%CI: 2.22–5.47, *R*^*2*^ = 2.60] more responsive to lithium compared to those in the lowest PGS decile, and a linear relationship was observed across the various deciles [76]. Table 3 provides a summary of pharmacogenomics scores for lithium treatment outcomes.

**Table 3:** Summary of findings on the association between PGx-score and lithium treatment outcomes in patients with bipolar disorder.

Author	Medication studied	PGSs for	Target cohort and treatment outcome			Main association findings	
			*Cohort*	*N*	*Treatment outcome*	*Effect estimates [OR/RR (95% CI)]*	*R*^2^ *(%)*
Amare et al. [71]	Lithium	SCZ	ConLi^+^Gen	2586	Response	First PGS_SCZ_ decile group vs tenth PGS_SCZ_ decile for favourable response: 3.46 [1.42-8.41]	0.80
Amare et al. [70]	Lithium	MDD	ConLi^+^Gen	2586	Response	First PGS_MDD_ decile group vs tenth PGS_MDD_ decile group for favourable response: 1.54 [1.18–2.01]	0.70–0.91
Schubert et al. [73]	Lithium	SCZ and MDD	ConLi^+^Gen	2283	Response	Higher decile group vs lower decile group for poorer response: 2.54 [1.91–3.08]	1.85
Cearns et al. [74]	Lithium	SCZ and MDD	ConLi^+^Gen	1034	Response	Not specified	12.1 for clinical linear and 13.7 for a non-linear random forest.
Coombes et al. [72]	Lithium	ADHD	ConLi^+^Gen	2510	Response	Higher and lower PGS_ADHD_ group for poor response: 0.86 [0.77,0.95]	0.18
Amare et al. [76]	Lithium	Lithium responsiveness	ConLi^+^Gen	2367	Response	Tenth Li^+^R_PGS_ decile vs first Li^+^R_PGS_ decile for favourable response: 3.47 [2.22–5.47]	1.90 for categorical and 2.60 for continuous outcomes
Millischer et al. [75]	Lithium	BMI and BUN	SWEBIC	2357	CL_Li_	Positive association between PGS_BMI_ with CL_Li_ and negative association between PGS for BUN with CL_Li_	~0.54

*PGx-score* Pharmacogenomic polygenic score, *PGS* Polygenic score, *SCZ* Schizophrenia, *MDD* Major Depressive Disorders, *MDE* Major Depressive Episode, *BD* Bipolar Disorders, *ADHD* Attention Deficit Hyperactivity Disorders, *OCD* Obsessive-Compulsive Disorders, *PGS*_*SCZ*_ PGS for SCZ, *PGS*_*MDD*_ PGS for MDD, *GS*_*BD*_ PGS for BD, *PGS*_*ADHD*_ PGS for ADHD, *ConLi+Gen* International Consortium of Lithium Genetics, *SWEBIC* Swedish bipolar cohort, *BUN* Blood Urea Nitrogen, *CL*_*Li*_ Total body lithium clearance, *Li+R*_*PGS*_ PGS for lithium responsiveness, *BMI* Body Mass Index, *SD* Standard Deviation, *NSA* No significant association, *NR* Not reported, *NA* Not applicable, *CI* Confidence Interval.

## Discussion

In psychiatry, pharmacogenomic scores (PGx-scores) are emerging as novel tools for predicting treatment outcomes such as response, remission, resistance, side effects, or hospitalization rates. While the bench-to-bedside translation of PGx-scores has not yet been achieved, a growing body of evidence indicates their potential clinical use for treatment personalization. In this systematic review, we describe the landscape of 53 PGx-score studies in clinical psychiatry. These PGx-scores have been developed either from genetic variants associated with psychiatric or medical diagnoses (the majority of studies); or from pharmacogenomic variants associated with treatment outcome phenotypes (a few recent studies). Findings from these studies showed that individual PGx-scores account for only a small amount of variance in treatment outcomes, thus, there is insufficient evidence to support their direct clinical transition. Any future efforts toward clinical implementation need to be complemented by additional clinical data and/or biological markers.

First, we found that over 90% of PGx-scores have been developed based on genetic variants of psychiatric or medical diagnoses (e.g., SCZ, MDD, BD, ADHD, coronary artery disease (CAD)) or phenotypes related to diagnoses (e.g., cognitive function, personality traits, educational attainment, CRP level, BMI). Among these, the PGS_SCZ_ has been most extensively studied and has consistently shown an association with pharmacotherapeutic outcomes across drug classes, including antipsychotics, antidepressants, and lithium, explaining as much as 3.2% of interindividual variability in some treatment outcomes [42]. The consistent association of the PGS_SCZ_ and treatment outcomes may be attributed to two factors. First, SCZ has a strong genetic basis with a heritability estimate of 80-85% [77] and it is possible that PGS_SCZ_ captures a substantial amount of the phenotypic variance of the disorder. Previous studies have shown a direct correlation between a higher phenotypic heritability and a better predictive power of PGS [78]. Second, SCZ GWASs are well-powered, including cases and controls of diverse ancestral background [79, 80], leading to more accurate PGSs [81]. The size of GWAS discovery samples has been associated with better accuracy and predictive power of PGSs [81]. For example, the Psychiatric Genomics Consortium (PGC in 2009) found that common genetic variants explained only 3% of the total variance in risk to SCZ in a sample of 3322 individuals with SCZ and 3587 controls of European ancestry [82]. In a follow-up study (in 2014) with expanded sample size and diversity (36,989 cases, 113,075 controls, multiple cohorts of East Asian ancestral background), the variance explained by PGS_SCZ_ substantially increased to around 18% [83, 84].

It is important to highlight that in most of the reviewed studies, high PGS_SCZ_ was associated with *poor* treatment response [33–35, 38, 42, 44, 45, 56, 71, 73, 74], more treatment resistance [36, 37, 40, 43, 54], more antipsychotic-induced side effects [31, 32, 41] or more psychiatric hospitalizations [39]. A notable exception was a positive association with lower symptom burden in SCZ patients treated with clozapine [38]. A possible explanation is that high polygenic loadings for SCZ may index individuals with a higher neurodevelopmental contribution to mental disorder aetiology. Neurodevelopmental hypotheses are well established in SCZ; for instance, excessive synaptic pruning is linked to complement system genotype [85]. Psychosis prodrome and onset [86, 87] and TRS [88] have been linked to reduced brain volume and connectivity. These ‘hard-wired’ brain characteristics may be more difficult to influence therapeutically through first-line (e.g., non-clozapine) pharmacological strategies [88].

The review also identified polygenic associations between cardiometabolic disorders [60, 61], personality traits [58], and treatment outcomes. Higher PGSs for CAD, obesity, and neurotic personality were associated with poor response to antidepressants [58, 61], while a positive association was found with the PGS for openness personality [58]. This is possibly due to shared biological mechanisms, for example, a genetic overlap between major psychiatric disorders and cardiometabolic diseases [89–92], neuroticism [93], or openness personality traits [94] and also associated multimorbidity across these disorders [95] that might impact patients’ treatment outcomes. Personality traits have an impact on medication adherence, with neuroticism linked to non-adherence and openness to compliance [96]. These findings indicated that disease-related PGSs may help us understand underlying pathology and identify drug targets.

Second, from our review, it is clear that there is a major research gap regarding PGx-scores developed from pharmacogenomic variants [54, 62, 68, 69, 76]. The lack of these studies is associated with the limited availability of well-powered GWAS summary statistics on treatment outcomes (target sample) and challenges to collecting genetic and clinical data from patients of specific diagnoses treated with similar medications (discovery sample). Currently, large-scale GWASs leverage biobank datasets, where there is limited phenotyping on medication and missing standardized data on treatment outcomes. Although the current cohort sizes for PGx-score development are much smaller than those of large-scale diagnosis-based GWASs, promising initiatives are underway to achieve deeper phenotyping for medications such as lithium [97], clozapine [37, 98], and antidepressants [56]. For instance, the ConLi^+^Gen cohort, which aimed to study the genetics of lithium treatment response in individuals with BD, currently has a sample size of 2367 patients of European ancestry and 220 patients of Asian ancestry with current efforts underway for a larger more diverse cohort and more detailed phenotyping [97]. By expanding current efforts, there may be opportunities to develop PGx-scores with improved accuracy for clinical use.

The third finding from this review is that the PGx-score alone falls short of explaining adequate variance in treatment outcomes for clinical translation. Notably, the highest reported explained variance solely attributed to PGx-score, by leveraging genetic variants of TRS and BMI, was 5.6% in resistance to clozapine. To address this shortfall, the combination of PGx-scores with clinical data could potentially enhance clinical use. For instance, a study modelled PGS_SCZ_ + PGS_MDD_ with patients’ clinical characteristics using machine learning, was able to explain 13.7% of the variance in lithium treatment response [74]. A further example is a multimodal model combining PGS with sociodemographic, clinical, biomarkers and structural imaging to predict rehospitalization risk showed a negative predictive value of 81.57% compared with a PGS-only model (54.83%) [99]. Similarly, a study that modelled polygenic scores of SCZ, MDD, and BD, along with proxy DNA methylation data and clinical symptom variables showed good regression performance for the prediction of response to multiple antipsychotic drugs (ROC = 0.87 [95% CI: 0.87–0.88]) [35]. In patients with type 2 diabetes, combining PGS with clinical data such as smoking status, BMI, blood lipid levels, blood pressure, and the use of anti-hypertensive and lipid-lowering medications substantially improved the accuracy in classifying individuals into low-, moderate-, and high-risk categories for cardiovascular events to 83%, whereas accuracy was 58% with PGSs alone (29 optimized univariable PGS) [100]. It is evident from these studies that PGx-score can be clinically useful if prediction models are refined based on a combination of PGx-scores and clinical data.

## Limitations

Some of the limitations of the present systematic review should be highlighted. First, the study participants of the included studies were predominantly drawn from European populations which limits the ability to apply the study’s conclusion to non-European populations and raises concerns about the generalizability of the findings to more diverse populations. Second, the inconsistent reporting of the polygenic model parameters across studies makes it challenging to compare PGx-score in predicting pharmacological treatment outcomes. Third, a significant portion of the included studies lack sufficient statistical power to draw conclusive results to the broader populations. Finally, the lack of a standard definition of pharmacological treatment outcomes, differences in participants’ characteristics, and the use of multiple medications across the different studies make it difficult to compare findings and to perform meta-analysis.

Where associations between PGx-scores and treatment outcomes were established, effect size estimates (betas, odds ratios, hazard ratios) and measures of explained variance (*R*^*2*^) varied widely. For instance, the *R*^*2*^ of PGx-score models for predicting resistance to clozapine treatment with PGS_SCZ_ in TRS individuals ranged from 2.03% [37] to 5.62% [54]. Similarly, the reported odds ratios for clozapine response ranged from 1.94 [95%CI: 1.33–2.81] [38] to 6.50 [95%CI: 1.47–28.80] [36]. These inconsistent findings can partly be explained by phenotypic heterogeneity, evident in diverse definitions and measurements of treatment outcomes and by differences in the sample size of these studies. As an example, the definition of TRS and TRD varies widely across studies [37, 43, 61, 101–104]. Achieving uniformity in phenotype characterization and harmonizing assessments across studies would help improve the reliability of the PGx-score in treatment outcomes.

Variations in sample size can also affect the size of individual study effect estimates and their statistical significance. Studies with small target or discovery samples have limited statistical power to detect significant associations. Choi et al. have demonstrated that in a discovery cohort of 100,000 samples, 200–500 samples in the target cohort are requisite to achieve 80% power for predicting traits across a spectrum of heritability estimates (*h*^*2*^:0.11–0.23) in polygenic models [105]. Recruiting a sufficiently large and well-characterized sample of uniformly treated individuals is a common challenge in PGx-score studies [81, 106].

## Future directions in pharmacogenomic scores research

While PGx-scores hold promise for predicting treatment outcomes, they currently account for only a small proportion of the variance in treatment outcomes. This systematic review highlights the lack of well-defined phenotypes and small sample sizes that limit our ability to adequately quantify the genetic complexity associated with medication response. In this context, the following future directions may improve the predictive capacity of the PGx-score and move us closer to their clinical utilization in psychiatry.

### Biologically informed pharmacogenomic scores

Previous PGx-score studies have been developed based on conventional polygenic modelling approaches, where the effect of genetic variants across the entire genome are aggregated, without taking into account the biological significance of these variants on the phenotype of interest [84, 107]. A biology-informed polygenic score (B-PGS) model was introduced very recently as a novel approach to improve both the predictive capability and biological meaning of polygenic scores, while also reducing sequencing costs [108, 109]. For example, in a study to predict psychosis, a pathway-specific PGS that was restricted to genomic locations within “nervous system development” and “regulation of neuron differentiation”, explained a variance of 6.9% in the risk of psychosis, outperforming the conventional PGS where genome-wide SCZ variants accounted for only 3.7% [110]. Biology-informed polygenic score potentially increases the polygenic signal-to-noise ratio by excluding variants with little association with pharmacogenomic outcomes and also enhances the clinical interpretability of polygenic models by focusing on specific molecular pathways [111]. There is emerging evidence elsewhere in medicine that B-PGS may be useful for the identification of new drug targets, for instance, in inflammatory bowel disease [112].

### Multi-trait pharmacogenomic score

By leveraging the genetic correlation between multiple phenotypes, the multi-trait PGS approach aggregates genetic information across traits with the aim to improve the prediction power of PGx-scores [113–115]. For example, in patients with BD, the polygenic scores of SCZ or MDD explained 0.80% [71] and 0.91% [70] of the variance in lithium response, respectively. Interestingly, combining the polygenic scores of SCZ and MDD resulted in a better model, with an explained variance of 1.85% in lithium treatment response [73], indicating that multi-trait PGS outperforms single-trait PGS.

### Combining multimodal data and machine learning optimization

Researchers have begun to combine PGS with other data modalities, for example, with clinical and imaging data to improve model accuracy [74, 116]. Machine learning methods are progressively being adopted for the analysis of multimodal or complex data comprising PGx-scores, socio-demographic, behavioural and clinical information [117, 118]. This approach, exemplified in a few studies included in our review [35, 74], holds promising results for clinical translation. Nevertheless, replication of these complex studies is lacking and interpretation of machine learning algorithms could be difficult for clinicians, potentially limiting their acceptance [119, 120]. To overcome this barrier, data scientists and clinicians need to collaborate at an early stage of model development to ensure that these models are not only clinically useful but also calibrated and valid for local conditions and easily understandable for end users [121–123].

### Validation of polygenic models

Given the complexity of pharmacogenomic models, current sampling issues and the associated risks of false discovery and poor generalizability across different populations, external replication and validation of these models is critical for future implementation [25, 124–126]. Only 26.4% of studies included in this systematic review employed external validation [34, 35, 37, 42, 44, 45, 54, 56, 60, 61, 68, 70, 76, 127].

### Multi-ancestry pharmacogenomic score

Nearly 90% of samples in the target and discovery cohorts of studies included in our systematic review were of European descent. Genetic variations and their effect on treatment outcomes can vary significantly among different populations. Given the complex pattern of linkage disequilibrium (short genetic regions) and the significant difference in the frequency of genetic variants between populations, the PGx-score constructed from one ancestral cohort may have a lower prediction in another cohort [124, 128, 129]. For instance, in cardiovascular medicine, a Brazilian-specific warfarin PGx-score used in a warfarin dosing algorithm was more accurate in Brazil than the one developed in the European population [130]. Conversely, polygenic models that incorporate information from ancestrally diverse populations, improve prediction performance, particularly in underrepresented non-European populations [131–135]. Diverse sampling is required to develop and validate more generalizable and transferable PGx-scores across diverse populations [84, 129]. These limitations hamper the translation of research findings into clinical practice and raise health disparity concerns. Thus, improving diversity in pharmacogenomic research is an essential step in creating polygenic models with broader applications.

### Clinical implications of pharmacogenomic score

**Fig. 4:**
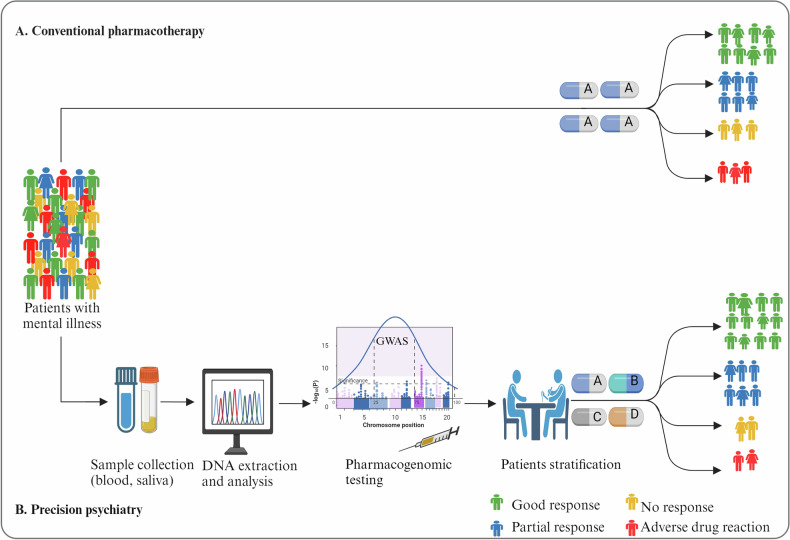
The potential use of pharmacogenomic scores in precision psychiatry. DNA Deoxyribonucleic acid.

While it is clear that further development is required to improve the accuracy of the PGx-scores, and alone they have low clinical utility, findings are advancing our knowledge of pharmacogenomics toward better personalization of treatment. For instance, the genetic loading for SCZ demonstrates some capability to stratify individuals based on lithium treatment response in BD [71, 73, 74] and clozapine dosage in individuals with TRS [34]. Drawing parallels from other disciplines, such as cardiovascular medicine, PGS for coronary artery disease has been used to reclassify patients from intermediate into high-risk categories translating into stronger statin use recommendations [136, 137]. Similarly, genome-wide PGS in cardiovascular research has identified individuals with a four-fold increased risk, prompting recommendations for aggressive cholesterol-lowering therapy [138]. Such evidence indicates that the polygenic scores have the potential to stratify patients, predict treatment outcomes, and inform therapeutic decision-making based on the genetic variation of population variation among different ancestral populations. Figure 4 shows the potential use of pharmacogenomic scores in precision psychiatry.

## Conclusions

In summary, this systematic review highlights that larger and more diverse target sample sizes, focussed on well-defined and standardized pharmacogenomic outcomes, with robust replication are required to optimize the development of PGx-scores. Currently, the variance explained by these models is too small for effective clinical translation. However, new techniques, such as B-PGS and the use of multivariate modelling combining multiple traits PGS with clinical data look promising to increase accuracy. Large-scale consortia focused on pharmacogenomics are required to improve sample size and diversity.

### Supplementary information

Complete search strategies for the association of pharmacogenomic polygenic scores and treatment outcomes in psychiatry practice

Detailed data extraction report in each study

Definition of treatment outcome for each study included in the review

Quality assessment result of included studies on the association between pharmacogenomic scores and treatment outcomes

## Data Availability

The original contributions presented in this study are included in the manuscript or supplementary tables. Further inquiries can be directed to the corresponding author.
