# Supplementary material for: Pharmacogenomic scores in psychiatry: systematic review of current evidence
Source: Transl Psychiatry. 2024 Aug 6;14:322. doi: 10.1038/s41398-024-02998-6 (PMC11303815; doi:10.1038/s41398-024-02998-6)
Supplement: Supplementary file 1 — Complete search strategies for the association of pharmacogenomic polygenic scores and treatment outcomes in psychiatry practice [file 41398_2024_2998_MOESM1_ESM.docx]

**Supplementary Table 1:** Complete search strategies for the association of pharmacogenomic polygenic scores and treatment outcomes in psychiatry practice

| Concepts | Search strings (PubMed) | PubMed | EMBASE | Web of Science |
| --- | --- | --- | --- | --- |
| Polygenic score (#1) | “Polygenic score*”[tiab] OR “Polygenic risk score*”[tiab] OR PRS OR “Risk profile score*”[tiab] OR “Genetic risk score*”[tiab] OR “Gene score*”[tiab] OR “Genetic score*”[tiab] OR polygenic*[tiab] OR "Pharmacogenomic Variants"[Mesh] OR "Pharmacogenomic Testing"[Mesh] OR Pharmaco-omic*[tiab] OR pharmacogeno*[tiab] OR "Pharmacogenetics"[Mesh] | 37,106 | 68,712 | 39,362 |
| Psychotropic drugs (#2) | "Antipsychotic Agents"[Mesh] OR "Antipsychotic Agents" [Pharmacological Action] OR antipsycho*[tiab] OR "Antidepressive Agents"[Mesh] Antidepress*[tiab] OR "Antidepressive Agents" [Pharmacological Action] OR "Anti-Anxiety Agents"[Mesh] OR Anti-Anxiet*[tiab] OR Antixiolytic*[tiab] OR Valproic acid[tiab] OR Valproate[tiab] OR Divalproate[tiab] OR Divalproex[tiab] OR Carbamazepine[tiab] OR Oxcarbazepine[tiab] OR Risperidone[tiab] OR Gabapentin[tiab] OR Lamotrigine[tiab] OR Licarbazepine[tiab] OR Pregabalin[tiab] OR Tiagabine[tiab] OR Zonisamide[tiab] OR Lithium[tiab] | 153,800 | 449,6049 | 385,309 |
|  | 1 # 2 | 1,005 | 1735 | 2149 |
|  | Studies included after-duplication | 3,303 | | |
|  | Studies included after title and abstracts | 127 | | |
|  | Records identified from citation searching | 6 | | |
|  | Studies included after full-text review | 53 | | |

**PubMed search string**

("Polygenic score*"[tiab] OR "Polygenic risk score*"[tiab] OR PRS OR "Risk profile score*"[tiab] OR "Genetic risk score*"[tiab] OR "Gene score*"[tiab] OR "Genetic score*"[tiab] OR polygenic*[tiab] OR "Pharmacogenomic Variants"[Mesh] OR "Pharmacogenomic Testing"[Mesh] OR Pharmaco-omic*[tiab] OR pharmacogeno*[tiab] OR "Pharmacogenetics"[Mesh] AND ((humans[Filter]) AND (english[Filter]))) AND ("Antipsychotic Agents"[Mesh] OR "Antipsychotic Agents" [Pharmacological Action] OR antipsycho*[tiab] OR "Antidepressive Agents"[Mesh] Antidepress*[tiab] OR "Antidepressive Agents" [Pharmacological Action] OR "Anti-Anxiety Agents"[Mesh] OR Anti-Anxiet*[tiab] OR Valproic acid[tiab] OR Valproate[tiab] OR Divalproate[tiab] OR Divalproex[tiab] OR Carbamazepine[tiab] OR Oxcarbazepine[tiab] OR Risperidone[tiab] OR Gabapentin[tiab] OR Lamotrigine[tiab] OR Licarbazepine[tiab] OR Pregabalin[tiab] OR Tiagabine[tiab] OR Zonisamide[tiab] OR Lithium[tiab] AND ((humans[Filter]) AND (english[Filter])))

**Embase (Ovid platform) search string**
(("Pharmacogenomic Variants" or "Pharmacogenomic Testing" or Pharmacogenetics or "Polygenic score*" or "Polygenic risk score*" or PRS or "Risk profile score*" or "Genetic risk score*" or "Gene score*" or "Genetic score*" or polygenic* or Pharmaco-omic* or pharmacogeno*) AND ("Antipsychotic Agents" or "Antidepressive Agents" or "Antidepressive Agents" or "Anti-Anxiety Agents" or antipsycho* or Antidepress* or Anti-Anxiet* or Valproic acid or Valproate or Divalproate or Divalproex or Carbamazepine or Oxcarbazepine or Risperidone or Gabapentin or Lamotrigine or Licarbazepine or Pregabalin or Tiagabine or Zonisamide or Lithium)).mp

**Web of Science**

TS=(("Pharmacogenomic Variants" OR "Pharmacogenomic Testing" OR Pharmacogenetics OR "Polygenic score*" OR "Polygenic risk score*" OR PRS OR "Risk profile score*" OR "Genetic risk score*" OR "Gene score*" OR "Genetic score*" OR polygenic* OR Pharmaco-omic* OR pharmacogene) AND ("Antipsychotic Agents" OR "Antidepressive Agents" OR "Antidepressive Agents" OR "Anti-Anxiety Agents" OR antipsycho* OR Antidepress* OR Anti-Anxiet* OR Valproic acid OR Valproate OR Divalproate OR Divalproex OR Carbamazepine OR Oxcarbazepine OR Risperidone OR Gabapentin OR Lamotrigine OR Licarbazepine OR Pregabalin OR Tiagabine OR Zonisamide OR Lithium))
