# Supplementary material for: Pharmacogenomic scores in psychiatry: systematic review of current evidence
Source: Transl Psychiatry. 2024 Aug 6;14:322. doi: 10.1038/s41398-024-02998-6 (PMC11303815; doi:10.1038/s41398-024-02998-6)
Supplement: Supplementary file 3 — Definition of treatment outcome for each study included in the review [file 41398_2024_2998_MOESM3_ESM.docx]

**Supplementary Table 3:** Definition of treatment outcome in each study included in the review

| Authors | Treatment outcomes | Definition of treatment outcomes |
| --- | --- | --- |
| Guo et al., (2023) | Treatment response to second-generation antipsychotics | Treatment response to antipsychotics evaluated by the PANSS reduction rate at the last follow-up, which can be calculated as (PANSS endpoint total score) divided by (PANSS baseline total score -30) X 100. Response group = PANSS reduction rate ≥ 50%) and nonresponse group = PANSS reduction rate < 50%. |
| Okhuijsen-Pfeifer et al., (2022) | Response to clozapine in symptom severity | Symptom severity was assessed by treating physicians or trained study raters using the CGI-S scale. Symptom severity was defined as a quantitative measure (CGI-S score) and symptom severity was defined as a binary measure (low vs. high symptom severity). Low symptom severity corresponded to a (converted) CGI-S score of 1 to 3 (‘normal’ to ‘mildly ill’) and high symptom severity corresponded to a (converted) CGI-S score of 4 to 7 (‘moderately ill’ to ‘among the most extremely ill patients’). |
| Santoro et al., (2018) | Response to risperidone | Response to risperidone treatment was defined as a > 50% reduction in baseline PANSS total score |
| Zhang et al., (2019) | Response to risperidone or olanzapine | Response in symptom reduction was defined as ≥50% reduction in total symptom scores (on either the BPRS or the PANSS from baseline to the 12-week follow-up |
| Li et al., (2018) | Response to lurasidone | Response measured as ΔPANSS-Total that is the difference between baseline and LOCF for those with at least one PANSS rating after baseline. Subjects within the 30th percentiles for greatest or least improvement in PANSS-Total (referred to as best and worst responders |
| Hettige et al., (2016) | Response to second-generation antipsychotics in prescribed dose | Dosage was standardized according to three standardization procedures: Chlorpromazine Equivalents (CPZe), Defined Daily Dose, and the percentage of the maximum dosage (PM%) according to the product monograph from the Compendium of Pharmaceutical and Specialties. |
| Kappel et al., (2023) | Clozapine dosage | Clozapine dosage was categorized as the probability of taking the highest clozapine dose within low (<300 mg/day), standard (300–600 mg/day), and high (>600 mg/day). |
| Lin et al., (2023) | Clozapine use | Clozapine use was defined as clozapine use at ≥1 of the three time points during follow-up. |
| O’Connell et al., (2023) | Treatment resistance to clozapine | TRS defined as all the individuals with TRS in these samples were prescribed clozapine after the failure of at least two trials of antipsychotics |
| Talarico et al., (2022) | Treatment resistance to second-generation antipsychotics | TRS was defined as patients had to be unresponsive to at least two antipsychotics used in monotherapy for a period of 4 to 6 weeks with appropriate doses of the drug and to present moderate to severe psychopathology and persistence of positive symptoms |
| Gasse et al., (2019) | Treatment resistance to clozapine | TRS was defined as the first occurrence of either clozapine initiation or hospitalization due to schizophrenia during antipsychotic treatment within 18 months after at least two periods of different antipsychotic monotherapies lasting at least 6 weeks each |
| Werner et al., (2020) | Treatment resistance to first and second-generation antipsychotics | TRS was defined as the history of treatment with clozapine or 2) two or more failed trials of antipsychotic treatment, each of at least six weeks duration and with therapeutic dosage. At least one of the antipsychotics had to be a second-generation antipsychotic |
| Pardinas et al., (2022) | Treatment resistance to clozapine | TRS was defined as patients had to be unresponsive to at least two antipsychotics used in monotherapy for a period of 4 to 6 weeks with appropriate doses of the drug and to present moderate to severe psychopathology and persistence of positive symptoms. |
| Wimberley et al., (2017) | Treatment resistance to clozapine | TRS was defined as the first occurrence of either clozapine initiation or hospitalization due to schizophrenia during antipsychotic treatment within 18 months after at least 2 periods of different antipsychotic monotherapy lasting at least 6 weeks each |
| Martin & Mowry, (2016) | Treatment resistance to clozapine | TRS was defined as following criteria: (i) two or more of delusions, hallucinations, disorganization, and negative symptoms; (ii) moderate or severe current global assessment of functioning; (iii) continuous course of illness; (iv) moderate to severe pattern of disease severity; (v) current antipsychotic medication at the time of assessment |
| Kowalec et al., (2021) | Treatment resistance to clozapine | TRS was defined as patients who redeemed a clozapine prescription OR having received antipsychotic polypharmacy (i.e., ≥2 antipsychotics) simultaneously for ≥ 90 days |
| Facal et al., (2022) | Hospitalization related to clozapine intake | Two main outcomes were analyzed referred to as “ever readmission” and “admission history.” Ever readmission was a dichotomic classification of patients in two groups, those with ever readmissions in a psychiatric unit and those with one or nonpsychiatric admission. Admission history was measured as a combination of number of admissions and length of hospitalization. An ordinal definition with four ordered levels was considered: 1, no admissions; 2, less than three acute mental care hospital admissions; 3, three or more acute mental care hospital admissions or ever mid-term stay admission, and 4, ever long-term stay admission |
| Mayen-Lobo et al., (2021) | Clozapine metabolic ratio | Refractory psychosis was defined as unresponsive to at least two previous antipsychotic treatments. The metabolic ratio is calculated as the ratio of an unmetabolized drug to its main metabolite, N-desmethylclozapine or norclozapine, in plasma samples and is optimally defined as approximately two. Clozapine dosage is a consensus that doses below 100 mg may be insufficient for patients to respond to, thus the standard dose is usually between 300 and 600 mg. |
| Blackman et al., (2022) | Change in cognitive symptoms related to first-generation antipsychotics | The neuropsychological battery was used to assess working memory and was administered with three levels of working memory load and a control task. Participants viewed a sequence of briefly presented, individual numbers (digits 1–4) on a video monitor and responded using a keypad. They were instructed to indicate: the currently presented number in the 0-back condition (sensorimotor control condition), the immediately previously presented number in the 1-back condition, the number two positions earlier in the sequence in the 2-back condition, and the number presented three positions earlier in the 3-back condition. The WASI provided estimates of verbal, non-verbal, and overall IQ. For the Category Fluency test (a measure of processing speed and semantic fluency), participants were given 1 min in which to rapidly verbalize exemplars in response to a category prompt. The Story Memory task served as one index of verbal learning and episodic memory; participants listened to a paragraph-length story and were asked to repeat the story, immediately and after a 20–30 min delay. The HVLT Revised served as a second verbal memory index. During three HVLT learning trials, participants heard and immediately recalled a list of words. They were also asked to recall the words after a 20–25 min delay and to recognize them from within a longer list of words (recognition trials). |
| Yoshida et al., (2023) | AIWG | Weight gain was assessed as change in weight from the baseline assessment, and severe weight gain is defined as >7% of change in weight from baseline |
| Muntane et al., (2023) | AIWG | Change in BMI was the difference BMI from the baseline to 3 and 12 months follow up of taking antipsychotics |
| Morgenroth et al., (2023) | Antipsychotics-induced OCD | OCS and OCD were measured with Y-BOCS in which a cutoff score of 8 was defined for OCS ≥ 8 and a cutoff score of 13 for OCD ≥ 13). |
| Segura et al., (2022) | Antipsychotic-induced metabolic dysregulation | Metabolic dysregulation measured with the serum blood level of glucose, triglycerides, total cholesterol, high-density lipoprotein, and low-density lipoprotein and BMI |
| Hommers et al., (2021) | QT interval measure while taking antipsychotics | The QT interval was calculated using Fridericia’s formula, which corrects the QTc interval using the cubic root of the RR interval for a wide range of heart rates contrary to Bazett’s formula using the square root of the RR interval |
| Lu et al., (2022) | QT interval measure while taking antipsychotics | QT interval prolongation was defined as the QTc interval prolongation after antipsychotic treatment (applied 10 ms as threshold). |
| Maciukiewicz et al., (2019) | AIWG | Weight gain was defined as a participant as a ‘weight-gainer’ if their weight increased by 7% or more from baseline to the last observation carried forward. |
| Lacaze et al., (2020) | Clozapine induced myocarditis | Myocarditis case is defined as cases meet clinical and diagnostic criteria for myocarditis, developing within 45 days of commencing clozapine |
| Pain et al., (2022) | Response to antidepressants | Two measures of antidepressant response were defined. Remission is a binary measure attained when a patient’s depression symptom score decreases to a pre-specified threshold for the rating scale. Remission thresholds for scales used in these studies were: MADRS ≤ 10, QIDCS ≤ 5, HAMD 17 ≤ 7, HAMD-21 ≤ 7, and BDI ≤ 9. Patients who did not reach these thresholds were classified as non-remitting. The quantitative measure of Percentage Improvement was calculated as 100*(baseline score – final score)/baseline score. |
| Ward et al., (2018) | Response to antidepressants | Response to SSRIs in the percentage of change score was defined as the percentage change in depression score from baseline at four weeks. This was calculated by subtracting the score at four weeks from the baseline and dividing this difference by the score at baseline. |
| Gendep Invesigators et al (2013) | Response to antidepressants | Antidepressant treatment response in clinical trials has been defined as a categorical (yes/ no) variable based on a predefined cutoff value on a rating scale at study exit (e.g., a HAMD score ≤7 defines remission) or a cutoff value on the relative improvement expressed as a proportion of severity score reduction from study entry (e.g., an improvement of ≥50% defines response) |
| Nøhr et al., (2022) | Response to vortioxetine | Patients were clinically diagnosed with MDD by DSM-IV. They were currently in a depressive episode of ≥3 months’ duration and had a MADRS total score ≥26 at the baseline visit. The primary outcome was the total improvement from baseline (change from baseline * (−1)) in depression symptoms measured by MADRS. |
| Amare et al., (2019) | Response to antidepressants | Response to SSRIs in MDD was defined after 4 weeks of SSRI treatment as a ≥ 50% reduction from baseline in the HRSD-17 or QIDS-C16. |
| Men et al., (2023) | Response/remission to antidepressants | Relapse and remission to sertraline and olanzapine were the outcome. Remission was defined as the absence of delusions and hallucinations and a HAMD17 total score ≤10 for two consecutive weeks. Relapse was defined as at least one of the following: (1) sufficient SCID-rated symptoms to meet criteria for a DSM-IV major depressive episode; (2) HAMD17 total score ≥18; (3) SCID-rated psychosis (delusions or hallucinations); or (4) other significant clinical worsening (i.e., suicidal ideation or attempt, development of mania or hypomania, or psychiatric hospitalization). |
| Garcia-Gonzalez et al., (2017) | Response to antidepressants | Continuous measure of improvement is calculated as the percentage change in symptom score and symptom remission. Percentage change was preferred to absolute change because it is less correlated with initial severity, relatively independent of the scale, and closely reflects clinician's impression of improvement. Remission was defined as a score below a consensus cut-off that corresponds to absence of depression for each scale |
| Li et al., (2020) | Response to esketamine | Esketamine treatment response outcome was assessed at the 4-week study endpoint using one continuous variable (percent change from baseline in the MADRS score) and two dichotomized variables (responder status, defined by a reduction of ≥ 50% on the MADRS, and remission status, defined by achieving a final MADRS score of < 12). |
| Tansey et al., (2014) | Response to antidepressants | Antidepressants treatment response was defined as a continuous variable reflecting proportional reduction in depression severity on the primary depression rating scale from baseline to the end of treatment, adjusted for age, sex and recruiting center/study. |
| Marshe et al., (2021) | Response to venlafaxine | Response was defined as HRSD score ≤7 in a meta-analysis of the IRL-GREY, STAR*D, and CANBIND-1 cohorts |
| Fanelli et al., (2022) | Non-response to antidepressants in symptom severity | Symptom severity was assessed through the MADRS. The treatment response group included patients with MDD who responded to their current antidepressant treatment and showed at least 50% improvement in symptom severity, while the non-response group included patients non-responding at least to one antidepressant treatment and classified as Stage I-IIII for Brescia, GSRD, and Tartu cohorts. Symptom severity was assessed using the QIDS- Clinician-rated scale (QIDS-C16) every two weeks. Response to current treatment/remission was defined as a 50% decrease in symptom severity and QIDS-C16 ≤5 at week 12, respectively for the STAR*D cohort. Response to the current treatment and remission was measured using the 21-it HAMD21) at six weeks (50% improvement from baseline and HAMD21 ≤7, respectively) in the Munster cohort |
| Guo et al., (2018) | Response to scopolamine | Response was defined as ≥ 50% change in MADRS score at day three from baseline |
| Meijs et al., (2022) | Response to antidepressants | The prediction analysis, first we focused on the dimensional improvement of depressive symptoms, and then on categorical improvement (response, defined as ≥50% reduction of baseline score measured by QIDS |
| Campos et al., (2022) | Chronic pain while taking antidepressants | For each antidepressant participants had taken, they were asked to rate its effectiveness using the item: ‘How well does/did [name of the antidepressant] work for you?’. The possible responses were as follows: ‘not at all well’, ‘moderately well’ and ‘very well’. We defined chronic pain according to the IASP as pain persisting or recurring for longer than 3 months |
| Zwicker et al., (2018) | Response to antidepressants | Treatment response was defined as the total score on the MADR, administered weekly for 12 weeks by trained psychiatrists and psychologists with high interrater reliability. |
| Amare, et al., (2018) | Response to antidepressants | Treatment response and remission to SSRIs, while treatment response was determined as a ≥50% reduction from baseline in the HRSD-17 or QIDS-C16 total scores, SSRI treatment remission was defined as achieving a HRSD-17 score ≤7 or a QIDS-C16 score ≤5 at 4 or 8 weeks of treatment. |
| Fanelli et al., (2021) | Response to antidepressants | Response was defined as a MADRS score < 22 and a decrease of at least 50% compared to the onset of the current MDD episode after at least four weeks of treatment. Non-responders were patients who did not respond to one antidepressant of adequate duration (at least four weeks) and dose during the current episode (possible response to following treatments was not assessed due to the cross-sectional study design). TRD was defined as a lack of response to at least two antidepressants of adequate duration (at least four weeks) and dose |
| Taylor et al., (2021) | Treatment resistance to antidepressants | TRD was defined as diagnosis of a primary affective disorder according to the International Classification of Diseases, 10th Edition (ICD-10) and failure to adequately respond to ≥1 antidepressant treatment trial during the current episode |
| Wigmore et al., (2020) | Treatment resistance to antidepressants | Treatment resistance to antidepressants was assessed in GS: SFHS using only individuals who had been prescribed at least one antidepressant at an adequate dose and duration and those individuals who had been prescribed more than two antidepressants. |
| International Consortium on Lithium, G., et al., (2018) | Response to lithium treatment | Lithium treatment response was assessed using the validated ALDA score. This scale quantifies symptom improvement over the course of treatment (A score, range 0–10), which is then weighted against five criteria (B score) that assess the quality of evidence for the response score, to arrive at a total Alda score. For dichotomized assessment of treatment response, patients with a total score of 7 or higher were categorized as good responders, and the remainder were categorized as poor responders. For continuous assessment of treatment response, Alda A scores were used. |
| Amare et al., (2021) | Response to lithium treatment | The same as in International Consortium on Lithium, G., et al., (2018) |
| Schubert et al., (2021) | Response to lithium treatment | The same as in International Consortium on Lithium, G., et al., (2018) |
| Cearns et al., (2022) | Response to lithium treatment | The same as in International Consortium on Lithium, G., et al., (2018) |
| Coombes et al., (2021) | Response to lithium treatment | The same as in International Consortium on Lithium, G., et al., (2018) |
| Amare et al., (2023) | Response to lithium treatment | The same as in International Consortium on Lithium, G., et al., (2018) |
| Millischer et al., (2022) | Lithium body clearance | The primary outcome of interest was the natural logarithm of total body clearance for lithium at each timepoint (CL_Li_ [log (L per day)]), calculated as the ratio between the daily lithium intake and serum lithium concentration, as defined by a steady-state pharmacokinetic model with a bioavailability of 100%. |

**Abbreviations:** PANSS: Positive and Negative Syndrome Scale; CGI-S: Clinical Global Impression-Severity; BPRS: Brief Psychiatric Rating Scale; LOCF: Last Observation Carried Forward; TRS: Treatment resistance schizophrenia; WASI: Wechsler Abbreviated Scale Intelligence; IQ: Intelligence quotient; HVLT: Hopkins Verbal Learning Test; BMI: Body Mass Index; AIWG: Antipsychotics-induced Weight Gain; OCS: Obsessive-compulsive symptom; OCD: Obsessive-compulsive disorders; Y-BOCS: Yale–Brown Obsessive–Compulsive Scale; MADRS: Montgomery-Åsberg Depression Rating Scale; QIDCS: Quick Inventory of Depressive Symptomatology; BDI: Beck Depression Inventory; HAM-D: Hamilton Depression Rating Scale; MDD: Major Depressive Disorders; DSM-IV: Diagnostic and Statistical Manual of Mental Disorders-fourth edition; SCID: Structural Clinical Interview for the DSM-IV; SSRIs: Selective Serotonin Reactive Inhibitors; HRSD: Hamilton Rating Scale for Depression; STAR*D: Sequenced Treatment Alternatives to Relieve Depression; IRL-GREY: Incomplete Response in Late Life Depression: Getting to Remission; CANBIND-1: Canadian Biomarker Integration Network for Depression Study; GSRD: Group for the Study of Resistant Depression; IASP: International Association for the Study of Pain; QIDS: Quick Inventory of Depressive Symptomatology; TRD: Treatment Resistance Depression; ICD-10: International Classification of Diseases, 10th Edition; GS: SFHS: Generation Scotland: the Scottish Family Health Study; ALDA score: Retrospective Criteria of Long-Term Treatment Response in Research subjects with bipolar disorder; CL_Li_: Lithium body clearance
