# Supplementary material for: Pharmacogenomic scores in psychiatry: systematic review of current evidence
Source: Transl Psychiatry. 2024 Aug 6;14:322. doi: 10.1038/s41398-024-02998-6 (PMC11303815; doi:10.1038/s41398-024-02998-6)
Supplement: Supplementary file 4 — Quality assessment result of included studies on the association between pharmacogenomic scores and treatment outcomes [file 41398_2024_2998_MOESM4_ESM.docx]

**Supplementary Table 4:** Quality assessment result of included studies on the association between pharmacogenomic scores and treatment outcomes

| Author (year) | Q1 | Q2 | Q3 | Q4 | Q5 | Q6 | Q7 | Q8 |
| --- | --- | --- | --- | --- | --- | --- | --- | --- |
| Yoshida et al., (2023) | Y | N | Y | N | Y | Y | Linear and logistic regression | Y |
| O’Connell et al., (2023) | Y | N | Y | Y | Y | Y | Chi-square | Y |
| Muntane et al., (2023) | Y | N | Y | N | Y | Y | Multiple linear regression and chi-square | Y |
| Morgenroth et al., (2023) | Y | N | Y | N | Y | Y | Linear and logistic regression | Y |
| Lin et al., (2023) | Y | N | Y | N | Y | Y | Multinomial logistic regression | Y |
| Kappel et al., (2023) | Y | Y | Y | Y | Y | Y | Binomial and multinomial logistic regression | Y |
| Men et al., (2023) | Y | N | Y | N | Y | Y | Logistic regression | Y |
| Guo et al., (2023) | Y | N | Y | Y | Y | Y | Machine learning analysis | Y |
| Amare et al., (2023) | Y | N | Y | Y | Y | Y | Linear and logistic regression | Y |
| Talarico et al., (2022) | Y | N | Y | N | N | Y | Logistic regression analysis | Y |
| Segura et al., (2022) | Y | N | Y | N | Y | Y | Linear mixed effects | Y |
| Pardinas et al., (2022) | Y | Y | Y | Y | Y | Y | Logistic regression analysis & Meta-analysis | Y |
| Pain et al., (2022) | Y | Y | Y | Y | Y | Y | Linear regression and meta-analysis | Y |
| Okhuijsen-Pfeifer et al., (2022) | N | N | Y | N | Y | Y | Logistic regression analysis | Y |
| Nøhr et al., (2022) | N | N | Y | N | Y | Y | Linear regression | Y |
| Millischer et al., (2022) | Y | N | Y | N | Y | Y | Linear mixed effect | Y |
| Meijs et al., (2022) | Y | N | Y | Y | Y | Y | Linear regression | Y |
| Lu et al., (2022) | N | N | Y | N | Y | Y | Linear and logistic regression | Y |
| Fanelli et al., (2022) | Y | Y | Y | N | Y | Y | Linear regression and meta-analysis | Y |
| Facal et al., (2022) | Y | N | Y | N | Y | Y | Logistic regression | Y |
| Cearns et al., (2022) | N | N | Y | N | Y | Y | Regularized linear (Ridge and Elastic-net and random forest analysis | Y |
| Campos et al., (2022 | Y | N | Y | N | Y | Y | Logistic regression | Y |
| Blackman et al., (2022) | N | N | Y | N | Y | Y | Linear regression | N |
| Taylor et al., (2021) | Y | N | Y | N | N | Y | Elastic-net logistic regression | Y |
| Schubert et al., (2021) | Y | Y | Y | N | Y | Y | Linear and logistic regression | Y |
| Mayen-Lobo et al., (2021) | Y | N | Y | N | Y | Y | Logistic regression | Y |
| Marshe et al., (2021) | Y | N | Y | Y | Y | Y | Linear and logistic regression analysis | Y |
| Kowalec et al., (2021) | N | N | Y | N | N | N | Logistic regression | Y |
| Hommers et al., (2021) | N | N | Y | N | Y | N | Logistic regression | N |
| Fanelli et al., (2021) | Y | Y | Y | N | Y | Y | Regression analysis | Y |
| Coombes et al., (2021) | Y | N | Y | N | Y | Y | Linear and logistic regression | Y |
| Amare et al., (2021) | Y | N | Y | Y | Y | Y | Logistic and linear regression | Y |
| Wigmore et al., (2020) | Y | Y | Y | N | Y | Y | Linear mixed model analysis | Y |
| Werner et al., (2020) | N | N | Y | N | Y | Y | Logistic regression | Y |
| Li et al., (2020) | N | N | Y | N | Y | Y | Not specified | N |
| Lacaze et al., (2020) | N | N | Y | N | Y | Y | Logistic regression | Y |
| Zhang et al., (2019) | Y | N | Y | Y | Y | Y | Linear regression | Y |
| Maciukiewicz et al., (2019) | Y | N | Y | Y | Y | Y | Linear regression | Y |
| Gasse et al., (2019) | N | N | Y | N | Y | N | Hazard ratio | Y |
| Amare et al., (2019) | Y | N | Y | Y | Y | Y | Logistic regression analysis | Y |
| Zwicker et al., (2018) | Y | N | Y | N | Y | Y | Mixed effects model f | Y |
| Ward et al., (2018) | N | N | Y | N | N | Y | Random effects meta-analysis | N |
| Santoro et al., 2018) | Y | N | Y | Y | Y | Y | Linear regression analysis | Y |
| Li et al., (2018) | N | N | Y | Y | Y | Y | Logistic regression | Y |
| Guo et al., (2018) | Y | N | Y | N | N | Y | Linear regression analysis | Y |
| Amare, et al., (2018) | Y | N | Y | N | Y | Y | Logistic regression analysis | Y |
| International Consortium on Lithium, G., et al., (2018) | Y | N | Y | N | Y | Y | Linear and logistic regression and meta-analysis | Y |
| Wimberley et al., (2017) | Y | N | Y | N | Y | N | Logistic regression | Y |
| Garcia-Gonzalez et al., (2017) | Y | Y | Y | N | Y | Y | Linear regression analysis in each cohort, followed by a fixed effect meta-analysis | Y |
| Martin & Mowry, (2016) | N | N | Y | N | N | Y | Logistic regression analysis | Y |
| Hettige et al., (2016) | Y | N | Y | N | N | Y | Linear regression | Y |
| Tansey et al., (2014) | Y | Y | Y | N | N | Y | Linear regression analysis | Y |
| Gendep Invesigators et al (2013) | Y | Y | Y | N | N | Y | Linear and logistic regression | Y |

**Legends:**

**Y = Yea; N = No**

**Q1** = Was there a clear rationale for the selected PGS?

**Q2** = Was a power calculation performed?

**Q3** = Were the in- and exclusion criteria clearly described?

**Q4** = Was there an external validation cohort?

**Q5** = Was adjustment for multiple testing applied?

**Q6** = Are the methods used to construct the PGS specified?

**Q7** = What type of association analysis was used for the main comparison?

**Q8** = Were confounders or covariates considered in the analyses?
